# Coverage with the First Dose of Human Papillomavirus Vaccination among Females Aged 9–50 Years in Shenzhen, China: A Surveillance Based on Administrative Health Records in 2023

**DOI:** 10.3390/vaccines12010075

**Published:** 2024-01-12

**Authors:** Zian Lin, Xue Liang, Lixian Su, Weijun Peng, Hongbiao Chen, Yuan Fang, Siyu Chen, Weikang Yang, Wensheng Chen, Lijun Zhang, Zixin Wang

**Affiliations:** 1 Shenzhen Longhua District Maternity and Child Healthcare Hospital, Shenzhen 518000, China; linzian@lhfywork.com (Z.L.); yangweikang@lhfywork.com (W.Y.); chenwensheng@lhfywork.com (W.C.); zhanglijun@lhfywork.com (L.Z.); 2 Centre for Health Behavious Research, JC School of Public Health and Primary Care, The Chinese University of Hong Kong, Hong Kong SAR 999077, China; liangx77@link.cuhk.edu.hk (X.L.); chensiyu@link.cuhk.edu.hk (S.C.); 3 Shenzhen Futian District Maternity and Child Healthcare Hospital, Shenzhen 518000, China; xiandansumei@163.com; 4 Department of Epidemiology and Infectious Disease Control, Longhua Key Discipline of Public Health for the Prevention and Control of Infectious Diseases, Longhua Centre for Disease Control and Prevention, Shenzhen 518000, China; igeneral@126.com (W.P.); gesila2021@163.com (H.C.); 5 Department of Health and Physical Education, The Education University of Hong Kong, Hong Kong SAR 999077, China; lunajoef@gmail.com

**Keywords:** human papillomavirus, HPV, vaccination, vaccines, China

## Abstract

China started to offer human papillomavirus (HPV) vaccines to females aged 9–45 years in 2016. However, there was a lack of reports about HPV vaccination coverage in a representative sample of females in China. Therefore, this study aimed to examine the current HPV coverage and associated factors among females aged 9–50 years in Shenzhen, China, based on administrative health records kept by community health centers. A multistage random sampling approach was used. The research team randomly selected 18 community health centers in Shenzhen, and 3118 health records of females aged 9–50 years were then randomly selected from these health centers. Among all participants, 18.7% received at least one dose of HPV vaccination. The highest coverage was observed among females aged 18–26 years (23.4%), followed by those aged 27–35 years (22.0%) and 36–45 years (20.2%). Such coverage was very low among females aged 9–17 years (4.6%) and those aged 46–50 years (3.2%). Among females aged 18 years or above, higher education level, having a family doctor, and permanent residency in Shenzhen were associated with higher HPV vaccination coverage, while older age and being married/divorced were negatively associated with coverage. The HPV vaccination coverage in Shenzhen was 18.7% and there is a strong need for improvement.

## 1. Introduction

Human papillomavirus (HPV) is one of the most common sexually transmitted infections, which can affect the skin, genital area and throat [1]. By the age of 45 years, about four in five males and females will have experienced at least one episode of HPV infection [2]. Most HPV infections can be cleared by the body [3], but in 10–20% of females, these infections remain persistent [4]. Persistent infection with high-risk HPV has been proven to be the cause of cervical cancer and is associated with other cancers such as vulva, vagina, mouth/throat, penis and anus [5]. Globally, cervical cancer ranks as the fourth most common cancer affecting women [5], and China represented about 18% of global cases and 17% of global deaths [6]. In China, cervical cancer is the sixth most frequent cancer among all women [7], and the third most common cancer among women aged 15–44 years [8]. Cervical cancer cases and deaths have been increasing over the past 20 years [9]. In China, about 58,000 females were diagnosed with cervical cancer and 20,000 died from the disease in 2005 [10], and these two figures increased to 109,741 and 59,060, respectively, in 2020 [11].

HPV vaccination is highly effective in preventing cervical cancers and it is safe for females [12,13]. Currently, three types of HPV vaccines are available including bivalent, quadrivalent, and 9-valent vaccines [14]. All three vaccines protect against HPV 16 and 18, which together cause about 70% of all cervical cancers [15]. Women aged 9–45 years are recommended to receive a HPV vaccination [14], and the World Health Organization (WHO) has set a target aiming to have 90% of girls fully vaccinated against HPV by the age of 15 years in 2030 [3]. The European Union (EU) also calls for vaccinating at least 90% of the EU’s target girl population, and significantly increasing the vaccination rate among boys [16]. Men are also susceptible to HPV infection and have a low rate of seroconversion [17]. This highlights the significance of vaccinating males, which is still not widely practiced despite the extensive programs for females [17]. However, the HPV vaccination uptake rate was low in China. According to 2020 statistics, only 3% of Chinese females aged 9–45 years [18], and less than 1% of Chinese girls aged 9–14 years [19] received full HPV vaccination, which is far from the WHO targets [3]. In China, the implementation of HPV vaccination programs was delayed by about 10 years compared to Western countries, and HPV vaccination is not yet available for men. The HPV vaccination program in China started in August 2016 for females aged 9–45 years (hereafter referred to as “main scheme”) [20]. The main scheme first provided bivalent HPV vaccines to females aged 9–45 years from August 2016 [20], and then the quadrivalent HPV vaccine to the same age group of females from May 2017 [20]. The main scheme was further expanded to supply 9-valent HPV vaccine for females aged 16–26 years in April 2018 [21], and for females aged 9–45 years in August 2022 [22]. The cost of these HPV vaccines (three doses) ranged from CNY 1080 (USD 148 for the bivalent vaccine) to CNY 3993 (USD 547 for the 9-valent vaccine). To receive the HPV vaccination, females need to settle the payment first. Those who received bivalent and quadrivalent HPV vaccines would receive reimbursement (about 80% of the cost) from the basic health insurance (BHI) in China [23]. However, those who received the 9-valent HPV vaccine could not get reimbursement from the BHI. The BHI is the basic component of the Chinese healthcare insurance system. In 2020, the BHI covered over 95% of residents in China [24]. The insured residents need to settle the premium monthly and the BHI will cover all or most of the cost of basic medical services. To our knowledge, no commercial health insurance in China could cover the cost of HPV vaccination.

A pilot scheme under the national HPV vaccination program was launched in 2022 in some Chinese provinces for girls aged 13–15 years [25]. Shenzhen is one of the first pilot cities. The scheme is voluntary. Girls who signed up for the scheme can receive bivalent HPV vaccines without any cost. However, for girls who chose to receive quadrivalent or 9-valent HPV vaccines, they should follow the arrangement under the main scheme.

Few studies reported HPV vaccination coverage among females in mainland China [26,27,28]. In 2017–2019, only 2.83% of females aged 9–45 years in Shanghai, China, had received HPV vaccination [26]. Another study reported that 11% of female college students in mainland China received HPV vaccination in 2019 [27]. The increase in HPV vaccination coverage was relatively slow, as another study reported that 18.6% of mothers and 4.5% of daughters aged 9–14 years in Shanghai, China, had received HPV vaccination or made an appointment to receive it in 2022 [28]. However, the convenience sampling method was used in previous studies and selection bias may exist. There was a lack of reports about HPV vaccination coverage in a representative sample of females in mainland China.

To address the knowledge gap, we randomly selected administrative health records of females aged 9–50 years in Shenzhen, China, and retrieved information related to HPV vaccination uptake. This study also investigated the associations between socio-demographic characteristics and HPV vaccination uptake among the sub-groups of participants aged 9–17 years and 18–50 years.

## 2. Methods

### 2.1. Participants and Data Collection

The inclusion criteria were as follows: (1) females aged 9–50 years; (2) had lived in Shenzhen for more than six months; and (3) had established administrative health records in community health centers. Shenzhen is a city and special economic zone in the southern Chinese province of Guangdong, bordering Hong Kong to the south. Shenzhen is one of the four most developed cities (i.e., Beijing, Shanghai, Guangzhou and Shenzhen) in China, with a population of 17.68 million in 2021. In Shenzhen, the main scheme of the HPV vaccination program providing self-financed vaccines for females aged 9–45 years started in 2018. In 2022, the pilot scheme under the HPV vaccination program was initiated to supply free bivalent HPV vaccines for girls who meet the following three criteria: (1) under the age of 14 years; (2) registered in Guangdong schools; and (3) in the first year of junior high school. We excluded women aged over 50 years at the time of this study, as they could not benefit from the HPV vaccination implementation in China. Those who had migrated to other cities at the time of this study were also excluded from the study.

Since 2014, all Chinese residents who have lived in the same region for more than six months, regardless of age, permanent residency or whether they have a family doctor, should establish administrative health records in local community health centers [29]. The health records contain basic socio-demographic information, children development indicators, information related to pregnancy and childbirth (for females), chronic disease diagnosis and management, vaccination history, and whether the patients have a family doctor. In China, the family doctor services were initiated in May 2016. All people, regardless of age or residency, who have been living in the same city/town for more than six months are recommended by the local health authorities to register for the family doctor services. After registration, one family doctor will be assigned to each person to provide individualized health services including basic medical services, public health services, health management, health education and consultation, diagnosis and treatment, appointment scheduling, referrals, and home care services. All services are free of charge. Since the family doctor service is still in its early stage in China and registration for the services is voluntary, it is common that some women are hesitant to register.

The multistage random sampling approach was used. Eighteen community health centers in Shenzhen were randomly selected. The number of administrative health records of female residents aged 9–50 years was 320,000 (ranging from 7600 to 33,000 in difference centers). The research team randomly selected 1% of health records (*n* = 3200). After excluding 82 participants who had migrated to other cities at the time of this study, the final sample size for data analysis was 3118. Ethical approval was obtained from the Shenzhen Longhua District Maternity & Child Healthcare Ethics Committee (ref: 2022122201).

### 2.2. Sample Size Planning

Based on the formula for simple random sampling ($N=\mathrm{deff}\times \left(t^{2}\times \mathrm{PQ}\right)/d^{2}$), we assumed 25% of females received HPV vaccination in Shenzhen. With a t value of 1.96, d of 0.1P, and deff of 2, the target sample size was 2300. Assuming 30% of the female residents with administrative health records in Shenzhen have migrated to other cities at the time of the study, we needed to retrieve 3200 records to meet the target sample size.

### 2.3. Measures

#### 2.3.1. Background Characteristics

For adult participants, information on their age, education level, employment status, relationship status, ownership of an apartment, whether they have a family doctor, and permanent residency in Shenzhen was retrieved from the administrative health records. For participants who were 9–17 years, the socio-demographic information available in the health administrative system included whether they have a family doctor, and permanent residency in Shenzhen.

#### 2.3.2. Information about HPV Vaccination

The available information about HPV vaccination in the administrative health records included the following: (1) whether they have received at least one dose of HPV vaccination, (2) the types of HPV vaccination they received, (3) completion of HPV vaccination (received all required doses), and (4) the time of receiving the first dose of HPV vaccination. All this information was retrieved.

### 2.4. Statistical Analysis

Details related to HPV vaccination uptake (i.e., uptake of at least one dose of HPV vaccination, types of HPV vaccination received by them, whether they completed HPV vaccination, and year of receiving the first dose of HPV vaccination) among all participants and those of different age groups were presented. The frequency distribution of socio-demographic information among sub-groups of participants aged 9–17 years and 18–50 years was also presented. Using the patient receiving at least one dose of HPV vaccination as the dependent variable, univariate logistic regression models were used to investigate the associations between socio-demographic characteristics and the dependent variable among these two sub-groups of participants. Multivariate logistic regression models were fitted considering all variables with *p* < 0.05 in univariate analysis as candidates. Crude odds ratios (OR), adjusted odds ratios (AOR), and their 95% confidence intervals (CI) were obtained. Statistical analyses were carried out using R software (version 4.3.1, St. Louis, MO, USA). All tests were two-sided with *p* < 0.05 as statistically significant.

## 3. Results

### 3.1. HPV Vaccination Coverage

Among all participants, 18.7% received at least one dose of HPV vaccination. The highest coverage was observed among females aged 18–26 years (23.4%), followed by those aged 27–35 years (22.0%) and 36–44 years (20.2%). The lowest HPV vaccination coverage was observed among females aged 9–17 years (4.6%) and those aged 45–50 years (3.2%) (Table 1).

Regarding the types of HPV vaccination received by the participants, about half (51.2%) of the vaccinated participates received quadrivalent HPV vaccines, and fewer received bivalent (19.7%) or 9-valent (29.1%) vaccines. The majority of females aged 27–44 years received quadrivalent HPV vaccines (27–35 years: 63.4%; and 36–44 years: 75.5%). All vaccinated girls aged 9–17 years and 55.6% of females aged 45–50 years received bivalent HPV vaccines, and 85.3% of those aged 18–26 years received 9-valent vaccines. Among the vaccinated females, the HPV vaccination completion rate was 56.5% for bivalent HPV vaccines, 69.6% for quadrivalent HPV vaccines, and 68.8% for 9-valent HPV vaccines, respectively. Regarding the time of HPV vaccination, over half of the vaccinated participants (54.4%) received their first dose of HPV vaccines in 2022, while less than 10% of the women received their first dose before 2020 (5.8% in 2019 and 3.1% in 2018 or before) (Table 1).

### 3.2. Background Characteristics of the Participants

For participants aged 18–50 years, the majority of this sub-group of participants had a full-time job (66.7%), and were married/divorced (69.0%). Less than 20% of them owned an apartment (16.5%), had a family doctor (19.6%), and were permanent residents in Shenzhen (11.7%). Less than half of them attended tertiary education (41.5%). A small proportion of girls aged 9–17 years had a family doctor (22.7%) and were permanent residents in Shenzhen (33.1%) (Table 2).

### 3.3. Factors Associated with HPV Vaccination Uptake among Participants

The multivariate logistic regression models showed that among females aged 18–50 years, higher education level (senior high or equivalent: AOR: 2.01, 95% CI: 1.49, 2.72; college or above: AOR: 2.53, 95% CI: 1.88, 3.40; reference group: junior high or below), having a family doctor (AOR: 1.58, 95% CI: 1.24, 2.02), and being a permanent resident in Shenzhen (AOR: 1.49, 95% CI: 1.10, 2.01) were associated with a higher HPV vaccination uptake. Women who were older (45–50 years: AOR: 0.21, 95% CI: 0.10, 0.44) and married/divorced (AOR: 0.58, 95% CI: 0.44, 0.76) had a lower HPV vaccination uptake (Table 3).

In girls aged 9–17 years, no significant associations were found between having a family doctor (crude OR: 1.14, 95% CI: 0.30, 4.36) or being a permanent resident in Shenzhen (crude OR: 0.66, 95% CI: 0.17, 2.51) and the uptake of at least one dose of HPV vaccination (results not presented in the table).

## 4. Discussion

Our findings represented the latest estimate of HPV vaccination coverage among females in Shenzhen, China, which can inform service planning. This study was based on a random sample of administrative health records of Shenzhen residents with a relatively large sample size, which was its strength. The overall coverage of at least one dose of HPV vaccination was 18.7% among participants aged 9–50 years, which was lower than that of some developed countries (e.g., 53.6% in the United States in 2018 [30], and 38.6% in Korea in 2016 [31]). Some reasons might explain the difference in HPV vaccination coverage between China and these countries. First, the rollout of HPV vaccination programs was about 10 years later than in these countries. According to the Diffusion of Innovation Theory (DOI), it takes time for members of a social system to adopt an innovation (e.g., HPV vaccination) [32,33]. In China, the diffusion of HPV vaccination has not yet reached critical mass, the point at which enough females have received it so that the increase in HPV vaccination coverage becomes self-sustaining. Second, although females in China are free to choose the types of HPV vaccine, many prefer quadrivalent or 9-valent HPV vaccines [34]. Among vaccinated women, over half of them received their first dose of HPV vaccination in 2022 when 9-valent HPV vaccines became available [21,22]. However, the supply of these vaccines could not meet the demand. Females in China usually have to wait for at least six months before they can receive these vaccines [35]. Moreover, there are tangible barriers for females in China when making an appointment to receive the 9-valent HPV vaccines. Such appointment making requires facial recognition through a smartphone application, which is not easy for the users, especially for students aged under 18 years. Furthermore, unlike in many developed countries, out-of-pocket payment is involved for HPV vaccination in China [36,37]. The high cost of HPV vaccines, especially for the 9-valent vaccines, might be another barrier for females to receive such vaccination.

For girls aged 9–17 years who could benefit the most from the HPV vaccination, in our study, it was found that only 4.6% of them received HPV vaccination; such a rate is much lower than that in Western countries. The HPV vaccination rate had reached 36.4% in Europe and 53.4% in North America in 2014 [38]. In Australia, 80.2% of girls aged 15 years had received all three doses of the HPV vaccine in 2017 [39], and in Canada, the provincial/territorial HPV vaccination coverage ranged from 57.1% to 91.3% for girls in 2020 [40]. In 2022, the pilot scheme under the national HPV vaccination program started in some regions of China, including Shenzhen. The program provides free bivalent HPV vaccine for girls aged 13 years or above who study in secondary schools. One possible reason for the low uptake was that parents, who are the main decision makers of girls’ vaccination, may not be interested in the bivalent vaccines provided by the program [34]. The government should consider expanding the pilot scheme by covering quadrivalent or 9-valent HPV vaccines.

In line with previous studies [31,41,42,43], we found that adult participants with a higher education level had higher HPV vaccination coverage. People with a higher education level generally had better knowledge of HPV and HPV vaccination, which were facilitators of HPV vaccination [41,44,45]. HPV knowledge, such as the risk of HPV infection and the benefits of HPV vaccines, should be further disseminated, and a social media platform such as WeChat may be a good approach to convey HPV knowledge given its wide popularity in China. Having a family doctor was associated with higher HPV vaccination uptake in females aged 18–50 years. Family doctors in China play important roles in health education and health promotion. They might disseminate health communication messages promoting HPV vaccination for females during consultations. Being permanent residents in Shenzhen was also associated with a higher HPV vaccination uptake. Future programs should pay more attention to migrant populations, which accounted for nearly 70% of the residents of Shenzhen in 2022 [46]. Having a full-time job and owning an apartment represented a better economic status of the participants. Females aged 18–50 years with a better economic status might have lower barriers related to the cost of the HPV vaccines. Previous studies also showed that the income level played an important role in HPV-vaccination-related decisions [31,41,47]. Lowering the price of HPV vaccines may be helpful in promoting vaccination, and a discounted price of vaccination has been shown to be a facilitator [48]. Providing a subsidy for HPV vaccination may be helpful in promoting vaccination uptake. In the sub-group of girls aged 9–17 years, having a family doctor and permanent residency were not significantly associated with HPV vaccination uptake. One possible explanation was that parents or caregivers are key decision makers regarding HPV vaccination for girls aged 9–17 years. Therefore, the characteristics of the parents or caregivers, instead of those of the girls, would affect HPV vaccination among girls aged 9–17 years.

Our study had several limitations. First, the information available in the administrative health records was limited to some basic socio-demographic characteristics. This study was not able to cover some potentially important determinants of HPV vaccination uptake (e.g., knowledge and attitudes). For girls aged 9–17 years, the administrative health records did not include the characteristics of their parents or caregivers. Second, we conducted this study in Shenzhen, where the pilot scheme providing free bivalent HPV vaccines for girls was first implemented. Therefore, our findings could not be generalized to other Chinese cities without the free program. The coverage of HPV vaccination in other Chinese cities is expected to be lower. Furthermore, this was a cross-sectional study and could not establish causality.

## 5. Conclusions

The HPV vaccination coverage was 18.7% among females aged 9–50 years in Shenzhen, China. More attention should be paid to girls aged 9–17 years, who could benefit the most from HPV vaccination, as only 4.6% of them received HPV vaccination. For females aged 18 years or above, more efforts should be focused on promoting HPV vaccination among those who are of lower socio-economic status or internal migrants. Family doctors could play an important role in HPV vaccination promotion.

## Figures and Tables

**Table 1:** HPV vaccination coverage among females aged 9–50 years in 2023, in Shenzhen, China.

	9–17 Years	18–26 Years	27–35 Years	36–44 Years	45–50 Years	All Participants
**Uptake of at least one dose of HPV vaccination in different age groups**						
n/N, %	12/260, 4.6	150/642, 23.4	262/1189, 22.0	151/749, 20.3	9/278, 3.2	584/3118, 18.7
**Types of HPV vaccination received by participants in different age groups (among participants who had received at least one dose of HPV vaccination)**						
Bivalent, n/N, %	12/12, 100.0	7/150, 4.7	59/262, 22.5	32/151, 21.2	5/9, 55.6	115/584, 19.7
Quadrivalent, n/N, %	0/12, 0.0	15/150, 10.0	166/262, 63.4	114/151, 75.5	4/9, 44.4	299/584, 51.2
Nine-valent, n/N, %	0/12, 0.0	128/150, 85.3	37/262, 14.1	5/151, 3.3	0/9, 0.0	170/584, 29.1
**HPV vaccination completion by types of HPV vaccines (among participants who had received at least one dose of HPV vaccination)**						
Bivalent, n/N, %	3/12, 25.0	4/7, 57.1	39/59, 66.1	17/32, 53.1	2/5, 40.0	65/115, 56.5
Quadrivalent, n/N, %	0/0, 0.0	10/15, 66.7	119/166, 71.7	75/114, 65.8	4/4, 100.0	208/299, 69.6
Nine-valent, n/N, %	0/0, 0.0	87/128, 68.0	27/37, 73.0	3/5, 60.0	0/0, 0.0	117/170, 68.8
**Time of receiving the first dose of HPV vaccination (among participants who had received at least one dose of HPV vaccination)**						
2018 or before, n/N, %	0/12, 0.0	2/150, 1.3	9/262, 3.4	7/151, 4.6	0/9, 0.0	18/584, 3.1
2019, n/N, %	0/12, 0.0	4/150, 2.7	17/262, 6.5	10/151, 6.6	3/9, 33.3	34/584, 5.8
2020, n/N, %	2/12, 16.7	17/150, 11.3	44/262, 16.8	23/151, 15.2	2/9, 22.2	88/584, 15.1
2021, n/N, %	0/12, 0.0	38/150, 25.3	57/262, 21.8	30/151, 19.9	1/9, 11.1	126/584, 21.6
2022, n/N, %	10/12, 83.3	89/150, 59.3	135/262, 51.5	81/151, 53.6	3/9, 33.3	318/584, 54.4

**Table 2:** Background characteristics of the participants.

	18–50 Years		9–17 Years	
	*n*	%	*n*	%
Age, years				
18–26	642	22.5	-	-
27–35	1189	41.6	-	-
36–44	749	26.2	-	-
45–50	278	9.7	-	-
Education level				
Junior high or below	784	27.4	-	-
Senior high or equivalent	888	31.1	-	-
College or above	1186	41.5	-	-
Employment status				
Full-time	1905	66.7	-	-
Part-time/self-employed/unemployed/students	953	33.3	-	-
Relationship status				
Single	886	31.0	-	-
Married/divorced	1972	69.0	-	-
Owned an apartment				
No	2387	83.5	-	-
Yes	471	16.5	-	-
Had a family doctor				
No	2299	80.4	201	77.3
Yes	559	19.6	59	22.7
Permanent residency in Shenzhen				
No	2523	88.3	174	66.9
Yes	335	11.7	86	33.1

**Table 3:** Associations between background characteristics and human papillomavirus vaccination coverage among females aged 18–50 years in Shenzhen.

	Crude OR (95% CI)	*p* Values	Adjusted OR (95% CI)	*p* Values
Age, years				
18–26	1.00		1.00	
27–35	0.93 (0.74, 1.17)	0.52	1.22 (0.92, 1.61)	0.17
36–44	0.83 (0.64, 1.07)	0.15	1.28 (0.89, 1.82)	0.18
45–50	0.11 (0.06, 0.22)	<0.001	0.21 (0.10, 0.44)	<0.001
Education level				
Junior high or below	1.00		1.00	
Senior high or equivalent	2.42 (1.81, 3.25)	<0.001	2.01 (1.49, 2.72)	<0.001
College or above	3.63 (2.76, 4.77)	<0.001	2.53 (1.88, 3.40)	<0.001
Employment status				
Full-time	1.00		1.00	
Part-time/self-employed/unemployed/students	0.77 (0.63, 0.94)	0.01	0.83 (0.67, 1.02)	0.08
Marriage history				
Single	1.00		1.00	
Married/divorced	0.55 (0.46, 0.66)	<0.001	0.58 (0.44, 0.76)	<0.001
Owned an apartment				
No	1.00		1.00	
Yes	1.38 (1.10, 1.75)	0.006	1.06 (0.81, 1.38)	0.69
Had a family doctor				
No	1.00		1.00	
Yes	1.57 (1.27, 1.95)	<0.001	1.58 (1.24, 2.02)	<0.001
Permanent residency in Shenzhen				
No	1.00		1.00	
Yes	2.25 (1.76, 2.89)	<0.001	1.49 (1.10, 2.01)	0.01

Adjusted odds ratios, odds ratios obtained via multivariate logistic regression models considering all variables with *p* < 0.05 in univariate analysis.

## Data Availability

The data presented in this study are available from the corresponding author upon request. The data are not publicly available as they contain personal information.
